# Perceptions and Profiles of Young People Regarding Spa Tourism: A Comparative Study of Students from Granada and Aachen Universities

**DOI:** 10.3390/ijerph19052580

**Published:** 2022-02-23

**Authors:** Aida Pinos-Navarrete, Francisco Javier Abarca-Álvarez, Juan Carlos Maroto-Martos

**Affiliations:** 1 Department of Human Geography, University of Granada, 18071 Granada, Spain; jcmaroto@ugr.es; 2 Department of Urban and Spatial Planning, University of Granada, 18071 Granada, Spain; fcoabarca@ugr.es

**Keywords:** spa tourism, university students, young people, user segmentation, Self-Organizing Maps, Europe

## Abstract

Spa tourism has undergone important changes in recent decades, actively embracing wellness and wellbeing. However, this transition is taking place in different ways in Europe, and this has led to varying perceptions of thermalism that have little to do with its original conception. The main aim of this study was to analyse current perceptions of spa tourism amongst university students, so as to identify profiles and compare the differences between two study cases: Granada (Spain) and Aachen (Germany). For this purpose, we applied a methodology that combines artificial intelligence techniques with questionnaires containing both quantitative and qualitative variables. This enabled us to identify and characterize a series of profiles, so as to acquire detailed knowledge of the perceptions of these students regarding spa tourism in Granada and Aachen. On the basis of the results, the interviewees were grouped together into seven profiles from which we deduced that young Germans from Aachen visit spas more frequently and have a more realistic perception of the thermal sector than young Spanish people from Granada. This situation could limit present and future demand for spas in southern Spain. With this in mind, in this paper we present an updated assessment of the demand for spas amongst university students, in order to design effective geomarketing strategies in two cities with long spa traditions.

## 1. Introduction

The use of hot thermal waters and later spa tourism has a long history [1]. In the 19th century, the spa tourism sector became consolidated and began to spread from different cities in Western Europe to the rest of the continent [2,3,4]. Since then spa tourism has undergone important changes [5], shifting away from thermalism (i.e., the therapeutic use of hot water springs)—despite its proven therapeutic benefits—towards new emerging forms of tourism related with wellness [6,7,8]. These changes have coincided with an update of the subjective concept of human wellbeing, which is defined in many different ways in different parts of Europe [9,10]. This context of change is both a challenge and an opportunity for the spa sector [11,12], which Grob [12] defines as an up-to-date form of health tourism with extraordinary potential in the global context. These challenges require new segments of the market to be explored. One of the most important segments of the market is young people, who are potential clients in the short-, medium-, and even long-term. Within this framework, we focused particularly on young university students. This population group, who are well-educated and are soon to embark on potentially well-paid careers, are an attractive segment of the market to research as potential customers of spas. Furthermore, young people today often question established values and lifestyles. Many of them are more aware and more concerned about fitness and health (sometimes influenced by social networks) than in previous generations. University students in particular are becoming increasingly involved in movements such as “realfooding” or achieving a “balance between the body, mind and soul”. These new concerns are in line with the management philosophy of spas and with some of the changes they have been making to diversify the services they offer.

With this in mind, the initial hypothesis for this study is that due to its recent evolution, real and/or potential users of spa tourism (in this case, university students from two spa cities in different countries) may have a mistaken or perhaps distorted view of this sector. This widespread misunderstanding could obstruct the development of the sector in some European regions.

Numerous research studies have been conducted into the different perceptions of users, establishing profiles and analysing the geographic differences within the spa sector, resulting from the process of diversification and adaptation of the original concept of thermalism as a synonym for health. In Europe, these include the paper by Kelly [13] analysing the profile of visitors to retreats at a global level; a study of the perception of the characteristics of the geological surroundings of spas in Slovakia [14]; the investigations on spatial profiles of spa tourism establishments in Slovakia [15] and Hungary [16]; a study of the growth in demand for the hot springs sector in Rumania [17]; the research into motivational profiles in Serbia [18]; an analysis of the segmentation of the clients of spas in Poland in general [19,20], and in medical spas in Poland in particular [21]; the research conducted to identify a standard profile of spa clients in Portugal [8]; the analysis of profiles in relation to motivational factors with ANOVA in Slovenia [22]; and the studies analysing three clusters of clients in Greek spas [23,24]. Particularly in Spain, there has been research analysing the visitors to wellness centres in Gran Canaria [25], the perception of the quality of service and related forms of conduct [26], and recent analyses of sociodemographic profiles, and of profiles based on satisfaction and on the characteristics of stays at spas in Andalusia [27,28]. Outside Europe, spa tourism is also arousing interest and there have been various papers about user motivation and profiles. These include studies in Hong Kong [29,30], Taiwan [31,32], and those from South Korea [33] and the USA [34]. Other studies include the different treatments chosen by the users of spas in Ghana according to their sociodemographic characteristics [35], and a comparative study about the differing models of spas in Brazil and Portugal [36]. 

However, certain aspects of this subject have yet to receive sufficient attention in the scientific literature. For example, there are important differences in the perceptions, level of attachment and profiles of young people from different countries in relation to spa tourism at an international level [37]. We believe that a study that encompasses these different dimensions from a comparative perspective could make a valuable contribution to the research on this subject. For this purpose, we began by conducting a detailed analysis of the grey literature and the updated literature on our chosen subject. We discovered that although certain aspects of this question have already been explored in different ways, our line of research is original in the sense that it adopts a new approach to the assessment of current and potential users of spas, focusing in particular on young students from two cities as comparative study cases. It offers an interesting combination of public perceptions and scientific evidence, which is important for developing a broad understanding of a tourism sector that is currently undergoing a process of renovation [38]. The gathering of opinions and the identification of profiles could be helpful in the creation of successful policies and laws at a regional level for spa tourism in southern Spain, as indicated in recent research [39]. In addition, understanding the opinions of potential users could help the administration develop specific effective policies [40]. The study could also provide helpful guidance for creating a brand for a spa as a tourist destination, as has been done in the Baltic countries [41,42] or in four regions of central Europe [43]. This will also help with the design of a solid marketing strategy on social networks aimed more specifically at the segments of population that have the weakest attachment to this sector [44,45], in this case university students and young people in general. 

In this context, as part of the necessary analysis of perception, it is important to point out that the interpretation of surveys of opinion based exclusively on traditional statistical analysis can encounter certain difficulties resulting from the nature of the information and from the requirements of the available statistical methods. Techniques like ANOVA require compliance with certain criteria that cannot normally be met due to the nature of the information gathered from surveys of this kind [46]. These include normality of the data, independence and homoscedasticity. It is highly improbable that these criteria can be met given that a lot of survey data is gathered using Likert-type scales [47] with few levels. Other methodologies such as factorial analysis presuppose linear relations between factors and variables, and do not take non-linear relations into account [46]. However, there are certain methodological alternatives, such as the Artificial Neural Networks (ANN) paradigm, and particularly Self-Organizing Maps (SOM), which enable us to overcome these limitations. Self-Organizing Maps have proved to be more powerful than classic linear methods of analysis of the properties of variables and are especially useful due to the excellent visual or graphic representations they provide [48]. Recent researchers found that these methodologies were reliable for the analysis of qualitative surveys of opinion and Likert-type scales [49], although quantitative variables were not included in their study.

Therefore, the main objective is to analyse and compare the perceptions of university students regarding spa tourism in Granada (Spain) and Aachen (Germany). With this information we can identify profiles and establish comparisons between the two different geographical areas, so as to enable us to extract preliminary results and reflections that may be useful in the management of this sector in general terms. 

This objective can be achieved by establishing profiles for the participants in the survey and grouping them together by means of a Self-Organizing Maps type of neural network. Each profile is then characterized using statistical methods. In the case of the qualitative variables or questions from the questionnaire, we performed nonparametric Chi-squared type tests to obtain the Odds Ratio, as a means of evaluating the effect size of belonging to a particular profile and obtaining its equivalence with Cohen’s d. By contrast, for the quantitative variables we performed T-Student tests, measuring the effect size directly with Cohen’s d. Finally, a Heat Map visualization was obtained, which brought together all the tests and effect sizes for each profile/variable pair in one comprehensible image.

After presenting the recent dynamics and the general challenges affecting spa tourism in the first section of this article, in section two we present the context of the study; in section three we explain the methods used in this research; and finally, in section four we present the results of the case studies for the selected towns. The results are discussed in section five and the conclusions are set out in section six, where we will also present the consequences and the implications for the management of the spa sector of the results obtained in section four.

## 2. Study Context: Importance of a Comparative Study

Our broad study area for this research was Europe, within which we focused specifically on Germany and Spain. This study area was selected for two main reasons: Firstly, due to the fact that in some European countries, thermalism is an important segment of the tourism market with a high turnover and very satisfactory progression, while in other countries the sector is struggling; and secondly, due to the possibility of extrapolating the results to other regions and countries at an international level.

Central European countries, of which Germany is a perfect example, have rapidly adapted to this new model, which prioritizes wellness services. German spas have updated their facilities accordingly and have also developed very effective marketing strategies aimed at a younger target audience. However, in southern European countries such as Spain, this reconceptualization has not taken place in the same way, in spite of having a spa tradition that is just as old and important as in Germany. This is why in this research we decided, as case studies, to focus on Germany and Spain, prime examples of the different approaches towards spa tourism in Europe today. We also believe that it is important to establish, firstly, whether young people in these countries do indeed have different perceptions of spa tourism, and secondly to analyse whether these, often mistaken, perceptions are one of the reasons holding back the development and readaptation of spa tourism in Mediterranean countries.

Health tourism plays an important role in the German economy, in that there are over 300 spas which together account for 30% of the nights spent at German tourist destinations [50]. It provides direct or indirect employment for about 350,000 Germans and has an annual turnover of about US $36.4 billion [11]. These statistics show that thermalism is one of the most important sectors in the German tourism industry. For its part, Spain has a total of 112 spas in operation today [51], which receive almost a million water-takers a year (970,454 in 2019) and generate just over 3000 direct jobs, of which well over 50% are performed by women (2196 in 2019) [52].

However, in recent decades, the German health system has been under increasing financial pressure and various reforms have been introduced in order to guarantee fair and sustainable funding of the system [53]. As a result, spa treatment is no longer covered by the German national insurance system except in exceptional cases. These reforms have had a significant impact on the health and wellbeing tourism market and pose great challenges for the industry. However, they also offer new opportunities [11,54]. Germany continues to specialize in wellness, which has established itself as a new model for tourism that has been conceptually defined by authors such as Koncul [55] or Smith and Diekmann [56]. A new model for spas has arisen [57] in which traditional therapy based on taking the waters has become demedicalized and is now marketed as one of the many wellness products on offer [58]. This new model is now arriving in Spain, albeit with several years of delay, and with notable differences between the north and south of the country [59]. This delay could be exacerbated by COVID-19, despite the proven health benefits of taking the waters for those infected with the virus [60]. Generally, the users of Spanish spas are mostly elderly people and the duration of stay in these establishments is closely linked to age [61].

Within this framework, the regions selected in this study have many similarities in terms of the size of their population and its main characteristics (e.g., aging). In geographical terms, they also share similar relief, soils, landscape and traditional land uses. Their administrative, legal and planning frameworks also have many common features. As regards the history of the sector, Germany was one of the pioneering countries in the development of thermal tourism in Western Europe [62]. As a result, many of the most important processes and trends that define the sector first became consolidated in this central hub and only reached the rest of the spa towns in peripheral areas, such as Spain, many years later. This centre-periphery relationship continues to exist even today and the changes in the sector initiated in Central Europe take several years to spread to Mediterranean countries with a spa tradition such as Spain [63]. The spas in these countries are currently at a key turning-point in their Tourism Area Life Cycle [64], as they face up to the challenge of transforming traditional spa tourism. All of this while continuing to observe the pioneering countries, where this process of change is now in a more advanced phase, a fact that makes them particularly interesting for this research.

The German city of Aachen is a national point of reference in this field and there are records of the use of medicinal mineral water since the times of the Roman Empire. The name of the city is linked to its history as a spring. The name Aachen may derive from the Old German word “ahha” (water) and from the archaic “Aquis grani” used in the Middle Ages, which came from the Latin phrase, “Aquae granni”. The city flourished above all due to Emperor Charlemagne’s interest in the thermal waters. Convinced of their curative powers, he declared Aachen as his main palace of residence at the end of the eighth century [65]. For its part, Granada has a long tradition of thermal springs closely associated with its Arabic past. The province of Granada has one of the highest number of spas in Andalusia, some of them situated in towns whose names also refer to their long history as bathing sites, such as Alhama de Granada, whose name derives from the Arabic word “al-hamman” (bath, public baths).

Despite these similarities, there also seems a priori to be a significant gap between the “spa culture” of young people in Germany and in Spain. If this were true, it could have far-reaching consequences for the development of the Spanish spa sector. With this in mind, in this paper we conduct surveys of opinion to analyse whether in spite of the similarities between the two countries in terms of geography and thermal tradition, there are significant differences in the current perceptions of spas amongst young university students in Aachen (Germany) and Granada (Spain) as a preliminary starting point.

## 3. Materials and Methods

The step-by-step methodology took the following form:

1. Data Collection
  - Questionnaire preparation
  - Survey sample design
  - Reliability of instrument (questionnaire)
    - Preliminary experiment (reduced sampling)
    - Internal validation of questionnaire
    - Full experiment (complete sampling)
    - Internal validation of questionnaire
2. Data Analysis
  a. Dividing the respondents into groups
    - Self-Organizing Map Model (unsupervised)
    - Definition Number of profiles (supervised)
  b. Interpretation of the groupings
    i. Evaluation of statistically significance
      1. Continuous variables: T-Student test
      2. Categorical variables: Chi-squared test
    ii. Effect size as measure of importance
      1. Continuous variables: Cohen’s d (ES)
      2. Categorical variables: Odds Ratio, converted to ES

The above steps are described in detail in the following sections.

### 3.1. Data Collection

Data was collected using a semi-structured online survey translated into Spanish and German. Before designing the surveys, face-to-face conversations were held with students from the two universities to help identify the most suitable line of questioning for the online questionnaire. This work was also combined with in-depth interviews with the director of the spa in Aachen and with the managers of various spas in the province of Granada. During this initial field work, we also held informal conversations with other experts in the spa sector, who gave us additional insights into spa tourism. The survey consisted of a set of questions divided into four large blocks: (a) personal details of the respondents; (b) their knowledge of the spa sector; (c) their level of attachment to spas; and (d) their opinions regarding the characteristics of spas and of thermalism in their respective countries.

To obtain the data, we conducted a sample design which distinguished between the populations of students from the University of Granada (N = 60,000 students) and those from the University of Aachen (N = 50,000 students), some of whom were current users and others potential clients. To this end, the following formula was used:

$$n=\frac{N\times z^{2}\times p\times q}{d^{2}\times \left(N-1\right)+z^{2}\times p\times q}$$

In which:

*n*: the sample size

*N*: the total population in the study 

*z*: confidence level

*p*: prevalence

*q*: 1 − *p*

*d*: error; accuracy: 1 − *d*

As no previous studies have been conducted on this specific subject, the prevalence is unknown. An expected prevalence value of 50% was therefore taken as the most unfavourable value. As a result, the following data will be used:

Nuniversity of Granada = 60,000; Nuniversity of Aachen = 50,000

*z* = 95% ~ 1.96

*p* = 0.5

*q* = 0.5

In view of the initial estimate of prevalence of 50%, it was decided that the error should be less than 10% = 0.1 

Finally, in this survey a total of 1416 Spanish students were interviewed compared to 193 Germans. Given a confidence interval of 0.95, these sample sizes would imply an error of 2.77% for the Spanish sample group and 7.04% for the German.

It is also important to point out that a preliminary experiment in the form of a test was carried out to validate the questionnaire. In this test, internal validation of the instrument was performed, and the design was adjusted accordingly in order to obtain the best results. In the internal validation of the questionnaire, a Cronbach’s Alpha value of 0.858 was obtained, so demonstrating the reliability of the instrument, which scored well over the recommended ideal value of 0.7. 

### 3.2. Data Analysis

#### 3.2.1. Dividing the Respondents into Groups

For the identification of profiles, we used the methodology based on Self-Organizing Maps described in Abarca-Álvarez, Campos-Sánchez and Mora-Esteban [48,66] and especially in Abarca-Álvarez, Reinoso-Bellido and Campos-Sánchez [67]. Insofar as this methodology is based on an unsupervised learning technique, it enables us to create profiles or groupings without attributing definitions or meanings to them a priori, so reducing the great complexity of the data [68]. Once the artificial neural network has been assessed, the profiles can be obtained. The number of profiles is determined using an evaluation procedure that mixes quantitative information with non-statistical criteria, based on the expert knowledge of the analyst [69]. This hybrid method for calculating the number of profiles produces better results than strictly quantitative methods.

#### 3.2.2. Interpretation of the Groupings

After the SOM analysis, we identified a series of profiles. Each profile is normally characterized with basic statistics (average, standard deviation, maximum, minimum, etc.) in order to make it easier to understand [70]. This also enables us to assess the influence of each variable (or question in the questionnaire) in the make-up of the profiles that were obtained. To this end, and following the recommendations of the American Statistical Association [71], we quantified the statistical significance and the Effect Size (ES) for each variable–profile pair [72]. 

The statistical significance of the continuous numerical variables was evaluated using the bilateral Student’s *t*-test (*p*-value ≤ 0.05), while for the categorical variables, the non-parametric chi-square test was performed, with the necessary Yates’s correction [73]. Together with the statistical significance value, we also calculated the effect size (ES) [74], according to the Cohen’s d criterion in the continual variables and using the Odds Ratio (OR) in the categorical variables, thus verifying OR as suitable for binary variables [74] such as those presented in this study. As a means of comparing the ES and OR results, the Odds Ratio was converted into ES using the following formula ES = ln(OR)/1.81 [75].

ES is a measure of how the answers to each of the questions on the form are different and specific within the profile in question, compared to the overall values for the whole study population. For a proper interpretation of each variable, it is therefore important to bear in mind the importance of the ES.

On occasions a given variable, particularly if it is a categorical variable, can have a high ES in a given profile, without this necessarily meaning that this category is dominant in the profile in question. What this high ES is really highlighting is that the presence of this variable within this profile is significantly different from the way it appears or is distributed in the sample group as a whole. In other words, in categories with low frequency, if all or most cases of this category appear within one specific profile, the ES for this variable/profile will be very high, without this necessarily meaning that most of the items in this profile belong to said category. This explains why it is important to observe the ES in order to fully understand the significance of a particular profile, and also the relative or absolute frequencies, which is why these data are set out in the Table summarizing the profiles. 

As a unit of measurement of effect, ES is normally tabulated in four levels [74]:

- |ES| between 0 and 0.2 = Nil or Negligible Effect Size.
- |ES| between 0.2 and 0.5 = Small Effect Size.
- |ES| between 0.5 and 0.8 = Medium Effect Size.
- |ES| over 0.8 = Large Effect Size.

It is also important to bear in mind that the sign of the ES (positive or negative) informs you about the direction of the effect. A negative ES indicates that the effect decreases the mean, while a positive ES indicates that it increases the mean.

## 4. Results

**Figure 1:**
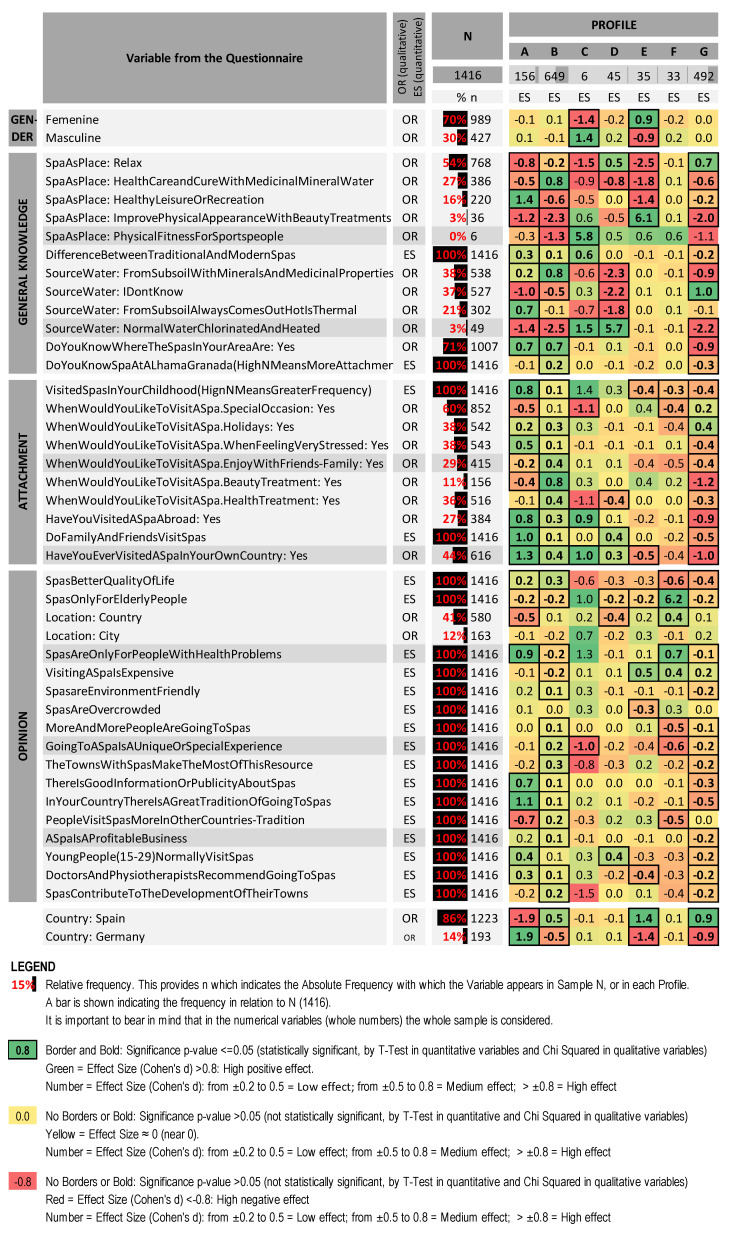
Characterization of the seven profiles identified (the profiles appear in the columns while the variables that characterize them appear in the rows). Created by the authors.

The main results of the analysis are presented in Figure 1 in the form of a heat map that summarizes the 1416 replies obtained in the survey. In this map the questions on the form are shown in the rows, while the seven profiles we identified appear in the columns. In the bottom part of the figure, we present the answers referring to nationality which were intentionally excluded from the construction of the model using SOM techniques, but which are included in the figure in order to ascertain whether there were any differences in the variables for each profile identified.

### 4.1. Profiles Identified

Once the profiles had been obtained, they were ordered according to the joint effect size of the variables belonging to the “Attachment Dimension”. This highlighted strong levels of attachment to spas in the first profiles (A and B) and gradually weaker levels as we moved down the list: 

#### 4.1.1. Profile A

Profile A represents 156 or 11.02% of the total number of 1416 interviewees. As regards the “General Knowledge Dimension”, it is important to point out that most of them viewed spas as places for healthy leisure and recreation (60% of interviewees ES = 1.4) and only 26% of them considered them as a place to relax (ES = −0.8). Up to 45% believed that the water came from the subsoil and that it was always hot (ES = 0.7). 88% knew that there were spas in their surrounding area (ES = 0.7). 

An “Attachment Dimension” can be observed in this profile with high scores for the different markers, indicating for example that they went to spas in their childhood (ES = 0.8), that they would go to a spa on a day when they felt stressed (ES = 0.5), that they had visited spas in foreign countries (ES = 0.8), that their family and friends went to spas (ES = 1.0) and that they had visited a spa in their country (ES = 1.3). As regards the “Opinion Dimension”, there are three opinions of particular note, namely that spas are not just for people with health problems (ES = 0.9), that there was good information or publicity about spas (ES = 0.7) and that there was a strong tradition of going to spas in their country (ES = 1.1).

Lastly, if we look at the variables that were excluded from the model, in this profile we saw a strong presence of respondents from the University of Aachen (Germany) (68% and ES = 1.9), with over half of all the Germans interviewed being classified within this profile.

#### 4.1.2. Profile B

This is the largest profile in terms of the number of interviewees with a total of 649 (45.83%). In general terms, there are hardly any notable differences between this profile and the most frequent overall answers. Perhaps the only difference worth highlighting was that the members of this profile answered slightly more frequently that they would go to a spa as a place for receiving beauty treatments (18% of the profile ES = 0.8), the vast majority of whom were Spaniards from the University of Granada (92% and ES = 0.5).

#### 4.1.3. Profile C

This group was very small, representing just six interviewees or 0.42% of the total sample group. Most of the group were males (83% and ES = 1.4), who regarded spas as a place for sportspeople to get fit (100% and ES = 5.8), highlighting differences between modern-day “spas” (with pools and jacuzzies with heated normal water and a range of wellness services) and traditional spas where people come to take medicinal mineral waters for therapeutic purposes (ES = 0.6). Some of them believed that the water used in traditional spas is normal water that is heated and chlorinated (ES = 1.5). They showed some kind of attachment to spas, stating that they had visited spas in their own country (83% and ES = 1.0) and abroad (67% and ES = 0.9). Nationality was not a differentiating factor in this profile.

#### 4.1.4. Profile D

45 of the 1416 interviewees (3.2% of the whole sample) were classified within this profile. Most considered spas as a place to relax (76% and ES = 0.5), and believed that the water used in spas is normal water that has been chlorinated and heated (100% and ES = 5.7). They stated quite often that their family and friends visit spas (ES = 0.4) and said that they had visited a spa in their own country (58% and ES = 0.3). They also answered that it was normal for young people to visit spas (ES = 0.4). Nationality was not a key differentiating factor in this profile.

#### 4.1.5. Profile E

This profile represents a total of 35 interviewees or 2.47% of the total sample group. Almost all the members were female (91% and ES = 0.9), and all of them considered that spas were places to improve your physical appearance with beauty treatments (100% and ES = 6.1), though few had actually visited a spa in their own country (23% and ES = −0.5). Many shared the opinion that going to spas was expensive (ES = 0.5), and relatively few said that doctors and physiotherapists recommended going to spas (ES = −0.4). Among the variables not included in the model, one could highlight the fact that all the members of this profile were Spanish (100% and ES = 1.4).

#### 4.1.6. Profile F

There were 33 interviewees in this profile or 2.33% of the total. Relatively low levels were observed in the attachment indicators, as few had visited spas in their childhood (ES = −0.3) and only 42% stated that they would visit a spa on a special occasion (ES = −0.4).

As regards the “Opinion Dimension”, most viewed spas as places for older people (ES = 6.2) and for people with health problems (ES = 0.7). They also stated more frequently than the mean for the whole group that spas are normally located in the country (58% and ES = 0.4) and that they are quite expensive (ES = 0.4). In the same way a relatively low number of interviewees in this profile regarded visiting a spa as a unique or special experience (ES = −0.6), or that more and more people are visiting them (ES = −0.5). Finally, it should be noted that nationality was not a key differentiating factor in this profile.

#### 4.1.7. Profile G

Profile G represents 492 interviewees or 34.74% of the total sample. A large number of these view spas as places for relaxing (72% and ES = 0.7), and a high percentage do not know where the water comes from (65% and ES = 1.0). There is a very low level of attachment, in that only 17% have visited a spa in their own country (ES = −1.0) and only 10% have done so abroad (ES = −0.9). Finally, only 2% said that they would visit a spa to receive a beauty treatment (ES = −1.2). 

In almost all the opinion-related questions, the members of this group presented below average scores. For example, relatively few of them believe that spas improve the quality of life (ES = −0.4), or that there is a strong spa tradition in their country (ES = −0.5). Amongst the variables not included in the model, one could highlight that this group contained a very high percentage of Spanish interviewees (students from the University of Granada) (96% and ES = 0.9). 

### 4.2. Detailed Results

**Figure 2:**
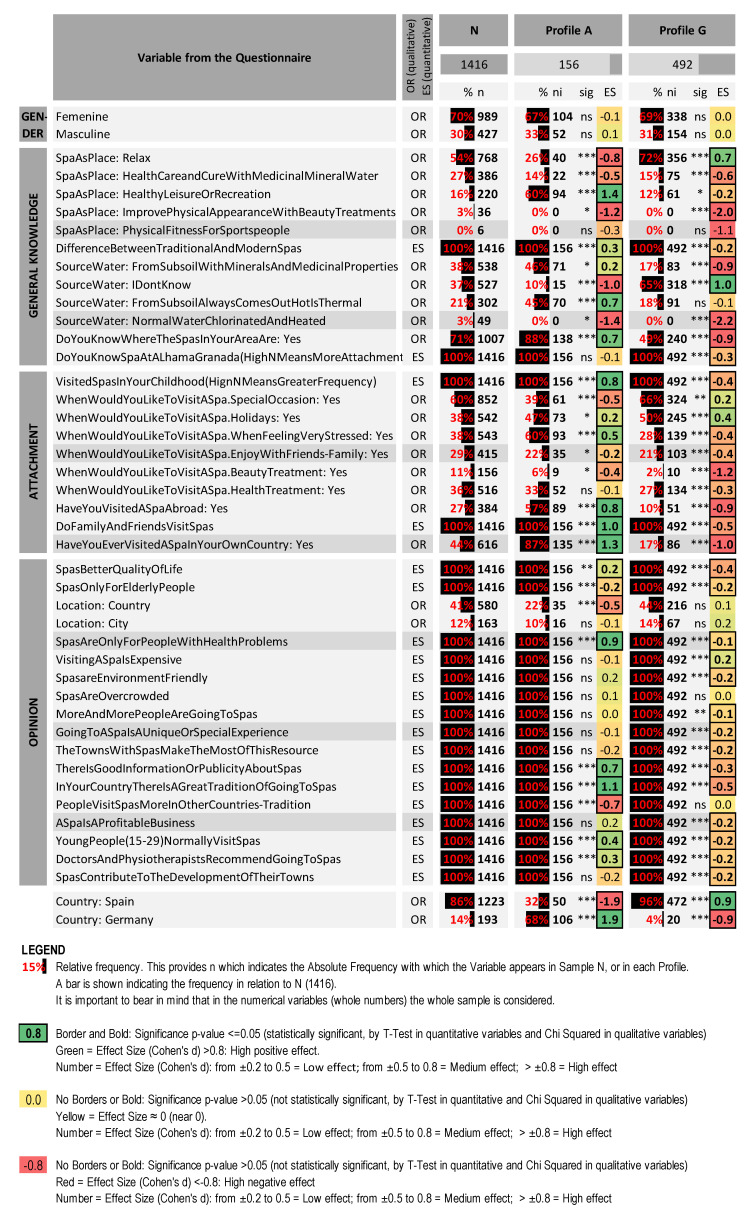
Detail of the most representative profiles—Profile A (mostly German university students) and Profile G (mostly Spanish university students). Sig: {ns = *p*-value > 0.05; * = *p*-value ≤ 0.05; ** = *p*-value ≤ 0.01; *** = *p*-value ≤ 0.001}. Created by the authors.

**Figure 3:**
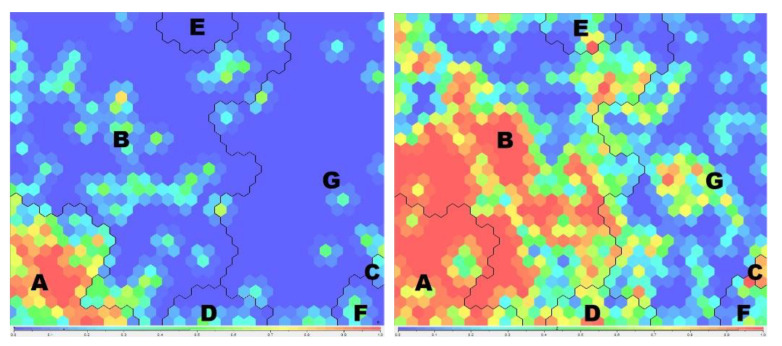
Self-organizing Maps (SOM) for the variable “Country of origin Germany” (**left**) and the variable “Have you ever been to a spa in your own country” (**right**). Letters represent the profiles name. Created by the authors.

Figure 2 shows the behaviour of the most significant variables and profiles in the analysis and Figure 3 shows the two SOM maps for the variable “Country of origin Germany” and the variable “Have you ever been to a spa in your country”.

Figure 3 clearly shows that, in the study cases, nationality is important in the organization of the SOM and in the definition of the profiles, even though this aspect was not included in the artificial intelligence model. It also shows that German university students (from Aachen) visit spas more often than Spanish university students (from Granada).

## 5. Discussion

Our review of the literature shows that, although research has been conducted on the profiles of spa clients, there are no specific studies of the perceptions of university students, especially from a comparative perspective between two cities with long spa traditions in different countries. In this study, we tried to cover what we believed was an important gap in the literature. In this way we confirmed our initial hypothesis that many university students have a distorted perception of spa tourism. It is also clear that the level of attachment and of knowledge about spas amongst young people from the University of Aachen is very much in line with the particular characteristics of the sector in their country. The students from the University of Granada, however, seem to know little about spas or their benefits. Similarly, their attachment to these establishments can be classified as very limited or practically non-existent, and is limited almost exclusively to very small groups of women who are looking for beauty treatments, or to select groups of sportspeople, generally professionals. In addition, and taking this interpretation a step further, the results show that as revealed by Dimitrovski and Todorovic [18], young students from Granada in particular have yet to grasp the full significance of spas, which they see as health centres for older people without establishing the connection between traditional spas and wellbeing. 

In previous studies with relevant results, the authors analysed the perceptions of tourists who visited spas in Poland and Serbia [18,20,21]. However, in order not to limit a priori the scope of our study and therefore the results, we decided to open the questionnaire both to students who had visited a spa at some stage and to those who had never been before and were therefore just potential clients. In this way we hoped to gather a wider range of opinions, rather than focusing exclusively on students who had visited these establishments and therefore already had a “spa culture” and a more realistic perception of the sector. 

In a similar way to previous researchers in other countries such as Poland [76], in this paper we analyse the perceptions of current or potential spa tourists in two cities and take a step further with the creation of profiles, so as to obtain a more graphic, more exhaustive understanding of the real situation. Authors such as Dimitrovski and Todorovic [18] or Dryglas and Salamaga [20] established two and three and profiles, respectively. However, given the rapid evolution of the sector in recent years and the sharp contrasts between countries [63] in terms of the need to cope with reforms in the national health systems and adapt to new trends in the conception of human wellbeing, we decided to establish seven profiles, so as to highlight the differences between the students we interviewed from Granada and Aachen in much finer detail. At the same time, we also wished to understand the geographical differences including the nationality variable, given that studies such as the one by Koh et al. [34] have already pointed out the differences between clusters centred on the gender and the level of education of the users. In this research, nationality stood out as a determining variable. In this way, we went beyond the studies based solely on sociodemographic characteristics in order to make a comparison between two cities in different countries, both of which have a long tradition of spa tourism, but which are currently evolving at very different rates. 

From a methodological point of view, this research takes a novel approach to identifying these new trends in thermalism by contrasting them in a comparative geographical analysis. These processes have never been discussed in previous research on the scale employed here, from a comparative perspective and with this particular methodology. Previous authors such as Dryglas and Salamaga [20] used K-Means cluster analysis, while in this research we used SOM techniques. SOM is a powerful alternative solution, which has enabled us to overcome some of the limitations associated with more traditional statistical methods [77]. It is also a reliable method for providing support for decision-making via the analysis and visualization of data [78]. Nowadays, SOMs are part of the toolbox available to statisticians seeking to analyse, represent and visualise data [79] and as such present certain operational advantages: (i) They facilitate the exploratory analysis of the data [68], providing visualisations that include all the original variables [46], with non-linear representations that are more powerful than the classic linear methods [48]; (ii) they provide more robust, more complete classifications than the traditional descriptive methodologies in general [80] and the k-means in particular [81], so enabling a visually effective exploration and validation [82]; and (iii) they provide powerful visualisations which are also easy to interpret [46], in that they maintain the topological relations of the data [48]. SOMs have been verified as a useful tool for analysing and visualising qualitative and quantitative data [83], and survey data in particular [84], assessing qualitative data using the Odds Ratio [67,85]. One of the main contributions of this research is that it integrates the above techniques into the analysis by converting them to the Cohen’s d Effect Size, so enabling us to compare their effect with that of the quantitative variables. 

Nonetheless, the use of SOMs can also present certain biases that must be taken into account. Certain conceptual errors can arise if the SOMs codify the survey data incorrectly, such as for example if categorical variables, such as gender, are codified as numerical variables, when in fact they should be assessed as dummy variables [48]. Another of the weaknesses of this methodology is that it is not always easy to integrate SOMs into decision-making processes [86]; and that the experts sometimes require a degree of creativity in order to be able to integrate the knowledge obtained [87].

In summary, and despite certain limitations, all the above points to the possibility of applying this method to other territories at an international scale, based on the establishment of a European framework and with the implementation of an ad hoc methodology that has been validated and is reproducible. This methodology provides new detailed results which can advance scientific knowledge in this field through a thematic line of research—thermalism—that has rarely been discussed in previous scientific literature. In future research, this methodology could be enhanced by including more variables, so as to make the profiles even more precise. It would also be interesting to analyse in greater depth the various factors that give rise to the different perceptions of spa tourism amongst German and Spanish university students, and to enlarge our sample size by conducting surveys in other places in the two countries. This would enable us to ascertain whether the perceptions vary greatly from one place to another due to different types of interest.

Like all industries, the spa sector must prepare for the future and find new clients amongst younger age groups. We are convinced that this methodology could be applied in other cities in different countries, as part of a bid to establish strategies for the dynamization and development of spa tourism that can address its current weaknesses.

## 6. Conclusions

In Granada (Spain) today, there are a large number of young university students, who could potentially be ideal clients of the spa sector due to their values, their lifestyle and their interest in health and personal wellbeing, but who paradoxically know very little about thermalism. Most of them have never or hardly ever visited spas in their city and/or country. If they have done so, it has generally been for beauty treatments (mostly females) or on brief weekend getaways for leisure or fun purposes, rather than in search of preventative health treatments and/or enhanced wellbeing. For their part, young German students from Aachen University visit spas more often than their Spanish counterparts. They frequently feel a strong attachment to the sector as they visited spas when they were children and the people in their immediate circle also visit them. They are very familiar with the concept of spa tourism, albeit in the form acquired in recent decades in Western Europe, in which spas are increasingly becoming places for leisure and relaxation rather than medical or health destinations.

The often distorted perceptions of spas amongst students from Granada must be taken into account, as they could have a negative limiting effect on the development of the spa sector in the south of Spain. Similar analyses of the perceptions of spas amongst potential young customers could be conducted in other parts of the country as part of the development of the sector in Spain, which has many underused medicinal mineral water establishments. This research, based on two case studies, has an original approach and methodology, which could be of practical value for the managers of spas in the Mediterranean region, when it comes to analysing the weaknesses in their supply and demand compared to other pioneering countries in the thermal sector, such as Germany. In addition, the different profiles identified in this study could be used as a basis for diversifying the range of products on offer in order to try to reach the widest possible target market. Within this context, geomarketing strategies could be designed in order to try to change the outdated, traditional attitudes towards spas amongst many young people today. Improving their knowledge of the sector could help promote a spa culture amongst young people in Spain in general and in Granada in particular.

In view of these results, the ultimate application of this research is to highlight the real situation of the demand for thermal tourism, so that Spanish spas in Granada can direct their efforts to correcting the stereotype images that many university students have of the spas in their region, as a means of boosting demand for these services. The aim would be to inspire new forms of organization that could enable spas from the two cites analysed to act as engines for the economic, social and environmental development of their surrounding geographical areas.

## Data Availability

The data presented in this study are available on request from the corresponding author.

## References

[1] Cohen M., Bodeker G., Cohen M. (2008). Spa Introduction. Understanding the Global Spa Industry: Spa Management.

[2] Jarrassé D. (2002). La Importancia del Termalismo en el Nacimiento y Desarrollo del Turismo en Europa en el Siglo XIX. http://hdl.handle.net/10810/37932.

[3] Van Tubergen A., Van der Linden S. (2002). A brief history of spa therapy. Ann. Rheum. Dis..

[4] Gianfaldoni S., Tchernev G., Wollina U., Roccia M.G., Fioranelli M., Gianfaldoni R., Lotti T. (2017). History of the baths and thermal medicine. Open Access Maced. J. Med. Sci..

[5] Smith M., Puczkó L. (2015). More than a special interest: Defining and determining the demand for health tourism. Tour. Recreat. Res..

[6] Mueller H., Lanz Kaufmann E. (2001). Wellness tourism: Market analysis of a special health tourism segment and implications for the hotel industry. J. Vacat. Mark..

[7] Erdeli G., Dincă A.I., Gheorghilaş A., Surugiu C. (2011). Romanian spa tourism: A communist paradigm in a post communist era. Hum. Geogr. -J. Stud. Res. Hum. Geogr..

[8] Gustavo Silva N. (2010). A 21st-century approach to health tourism spas: The case of Portugal. J. Hosp. Tour. Manag..

[9] Pierewan A.C., Tampubolon G. (2014). Spatial dependence multilevel model of well-being across regions in Europe. Appl. Geogr..

[10] Pyke S., Hartwell H., Blake A., Hemingway A. (2016). Exploring well-being as a tourism product resource. Tour. Manag..

[11] Pforr C., Locher C. (2012). The German spa and health resort industry in the light of health care system reforms. J. Travel Tour. Mark..

[12] Groß Matilde S. (2017). Gesundheitstourismus.

[13] Kelly C. (2012). Wellness tourism: Retreat visitor motivations and experiences. Tour. Recreat. Res..

[14] Chrobak A., Ugolini F., Pearlmutter D., Raschi A. (2020). Thermal Tourism and Geoheritage: Examining Visitor Motivations and Perceptions. Resources.

[15] Kasagranda A., Gurňák D. (2017). Spa and wellness tourism in Slovakia (A geographical analysis). Czech J. Tour..

[16] Jónás-Berki M., Csapó J., Pálfi A., Aubert A. (2014). A market and spatial perspective of health tourism destinations: The Hungarian experience. Int. J. Tour. Res..

[17] Surugiu C., Surugiu M.R., Mazilescu R. (2020). Social insurance system influence on spa tourism: Evidence for Romania. Anatolia.

[18] Dimitrovski D., Todorović A. (2015). Clustering wellness tourists in spa environment. Tour. Manag. Perspect..

[19] Dryglas D., Salamaga M. (2016). Applying destination attribute segmentation to health tourists: A case study of Polish spa resorts. J. Travel Tour. Mark..

[20] Dryglas D., Salamaga M. (2018). Segmentation by push motives in health tourism destinations: A case study of Polish spa resorts. J. Destin. Mark. Manag..

[21] Dryglas D., Różycki P. (2017). Profile of tourists visiting European spa resorts: A case study of Poland. J. Policy Res. Tour. Leis. Events.

[22] Rančić M., Pavić L., Mijatov M. (2014). Wellness centers in Slovenia: Tourists’ profiles and motivational factors. Turizam.

[23] Kamenidou I.C., Mamalis S.A., Priporas C.V., Kokkinis G.F. (2014). Segmenting customers based on perceived importance of wellness facilities. Procedia Econ. Financ..

[24] Papageorgiou M., Beriatos E. (2011). Spatial planning and development in tourist destinations: A survey in a Greek spa town. Int. J. Sustain. Dev. Plan..

[25] Medina-Muñoz D.R., Medina-Muñoz R.D. (2013). Critical issues in health and wellness tourism: An exploratory study of visitors to wellness centres on Gran Canaria. Curr. Issues Tour..

[26] Alén M.E., Rodríguez L., Fraiz J.A. (2007). Assessing tourist behavioral intentions through perceived service quality and customer satisfaction. J. Bus. Res..

[27] Anaya-Aguilar R., Gemar G., Anaya-Aguilar C. (2021). A Typology of Spa-Goers in Southern Spain. Sustainability.

[28] Anaya-Aguilar R., Gemar G., Anaya-Aguilar C. (2021). Factors associated with spa tourists’ satisfaction. Mathematics.

[29] Mak A.H., Wong K.K., Chang R.C. (2008). Health or self-indulgence? The motivations and characteristics of spa-goers. Int. J. Tour. Res..

[30] Denizci Guillet B., Kucukusta D. (2016). Spa market segmentation according to customer preference. Int. J. Contemp. Hosp. Manag..

[31] Chen J.S., Prebensen N., Huan T.C. (2008). Determining the motivation of wellness travelers. Anatolia.

[32] Chen K.H., Liu H.H., Chang F.H. (2013). Essential customer service factors and the segmentation of older visitors within wellness tourism based on hot springs hotels. Int. J. Hosp. Manag..

[33] Choi Y., Kim J., Lee C.K., Hickerson B. (2015). The role of functional and wellness values in visitors’ evaluation of spa experiences. Asia Pac. J. Tour. Res..

[34] Koh S., Jung-Eun Yoo J., Boger C.A. (2010). Importance-performance analysis with benefit segmentation of spa goers. Int. J. Contemp. Hosp. Manag..

[35] Adongo C.A., Amuquandoh F.E., Amenumey E.K. (2017). Modelling spa-goers’ choices of therapeutic activities. J. Hosp. Tour. Manag..

[36] Quintela M.M. (2011). Seeking ‘energy’vs. pain relief in spas in Brazil (Caldas da Imperatriz) and Portugal (Termas da Sulfúrea). Anthropol. Med..

[37] De la Hoz-Correa A., Muñoz-Leiva F., Bakucz M. (2018). Past themes and future trends in medical tourism research: A co-word analysis. Tour. Manag..

[38] Guodaar L., Bardsley D.K., Suh J. (2021). Integrating local perceptions with scientific evidence to understand climate change variability in northern Ghana: A mixed-methods approach. Appl. Geogr..

[39] Anaya-Aguilar R., Gemar G., Anaya-Aguilar C. (2021). Challenges of Spa Tourism in Andalusia: Experts’ Proposed Solutions. Int. J. Environ. Res. Public Health.

[40] Wang B., Tang H., Xu Y. (2017). Perceptions of human well-being across diverse respondents and landscapes in a mountain-basin system, China. Appl. Geogr..

[41] Smith M. (2015). Baltic health tourism: Uniqueness and commonalities. Scand. J. Hosp. Tour..

[42] Sziva I., Balázs O., Michalkó G., Kiss K., Puczkó L., Smith M., Apró E. (2017). Branding strategy of the countries in the Balkan region-focusing on health tourism. GeoJ. Tour. Geosites.

[43] Lebe S.S. (2006). European spa world: Chances for the project’s sustainability through application of knowledge management. J. Qual. Assur. Hosp. Tour..

[44] Sánchez Amboage E., Juanatey-Boga O., Valentín-Alejandro M.F. Los Medios Sociales, un nuevo escenario para la promoción turística Un análisis de los balnearios de Galicia más representativos en Facebook. Proceedings of the 10ª Conferencia Ibérica de Sistemas y Tecnologías de la Información (CISTI).

[45] Pinos A., Maroto J.C., Cejudo E. (2019). Turismo de salud y nuevas tecnologías en España: Facebook y Google Trends como herramientas de análisis. Crisis y Espacios de Oportunidad: Retos Para la Geografía: Libro de Actas.

[46] Tabrizi T.S., Khoie M.R., Sahebkar E., Rahimi S., Marhamatil N. (2017). Towards a patient satisfaction based hospital recommendation system. Appl. Sci..

[47] Likert R. (1932). A Technique for the measurement of attitudes. Arch. Psychol..

[48] Martín Guerrero J.D., Marcelli D., Soria-Olivas E., Mari F., Martínez-Martínez J.M., Soley Bech I., Martínez-Sober M., Scatizzi L., Gómez-Sanchis J., Stopper A. (2012). Self-Organising Maps: A new way to screen the level of satisfaction of dialysis patients. Expert Syst. Appl..

[49] Abarca-Alvarez F.J., Campos-Sánchez F.S., Mora-Esteban R. (2019). Survey assessment for decision support using self-organizing maps profile characterization with an odds and cluster heat map: Application to children’s perception of urban school environments. Entropy.

[50] Becker C. (2000). Freizeit und Tourismus in Deutschland–eine Einführung. Inst. Für Länderkunde (Hrsg.) Natl. Bundesrepub. Dtschl..

[51] AITB (2021). Mapa de Balnearios, Centros de Terapia y Centros Termolúdicos de España y Portugal. Asociación Iberoamericana de Termalismo y Bienestar. http://aitb.org.es/mapa.html.

[52] IGME (2019). Estadísticas de Balnearios en España. Instituto Geológico y Minero de España. http://aguasmineralesytermales.igme.es/ext/estadistica-ESP-balnearios.aspx.

[53] Pillmayer M., Scherle N., Pforr C., Locher C., Herntrei M. (2021). Transformation processes in Germany’s health resorts and spas–a three case analysis. Ann. Leis. Res..

[54] Cassens M., Hörmann G., Tarnai C., Stosiek N., Meyer W. (2012). Trend Gesundheitstourismus. Prävention Und Gesundh..

[55] Koncul N. (2012). Wellness: A new mode of tourism. Econ. Res. -Ekon. Istraživanja.

[56] Smith M., Diekmann A. (2017). Tourism and wellbeing. Ann. Tour. Res..

[57] Pinos A., Sánchez L.M., Maroto J.C. (2021). El turismo de balneario en Europa Occidental: Reconceptualización y nuevas funciones territoriales en una perspectiva comparada. Boletín Asoc. Geógrafos Españoles.

[58] Speier A.R. (2011). Health tourism in a Czech health spa. Anthropol. Med..

[59] Pinos A., Shaw G., Maroto J.C. (2020). Towards wellness? A case study of the profile of tourists visiting a southern Spanish spa. Int. J. Spa Wellness.

[60] Pinos A., Shaw G. (2020). Spa tourism opportunities as strategic sector in aiding recovery from COVID-19: The Spanish model. Tour. Hosp. Res..

[61] Esiyok B., Kurtulmuşoğlu F.B., Özdemir A. (2018). Heterogeneity in the determinants of length of stay across middle age and senior age groups in thermal tourism. J. Travel Tour. Mark..

[62] Peeters L., Houbrechts D. (2016). Spa Ville Thermale: Sources of the Spas.

[63] Smith M., Puczkó L. (2010). Taking your life into your own hands? New trends in European health tourism. Tour. Recreat. Res..

[64] Butler R.W. (1980). The concept of a tourist area cycle of evolution: Implications for management of resources. Can. Geogr..

[65] Imhof M. (2005). Aachen Dom-und Stadtführer, Broschüre.

[66] Abarca-Alvarez F.J., Mora-Esteban R., Campos-Sánchez F.S. (2018). Transparentar el conocimiento urbano para el apoyo a la decisión mediante inteligencia artificial: Comprendiendo la percepción infantil de los entornos escolares de Granada. Teknokultura.

[67] Abarca-Alvarez F.J., Reinoso-Bellido R., Campos-Sánchez F.S. (2019). Decision model for predicting social vulnerability using artificial intelligence. ISPRS Int. J. Geo-Inf..

[68] Spielmans S.E., Thill J.-C. (2008). Social area analysisss, data mining, and GIS. Comput. Environ. Urban Syst..

[69] Hair J.F., Black W.C., Babin B.J., Anderson R.E. (2009). Multivariate Data Analysis.

[70] Faggiano L., de Zwart D., García-Berthou E., Lek S., Gevrey M. (2010). Patterning ecological risk of pesticide contamination at the river basin scale. Sci. Total Environ..

[71] Wasserstein R.L., Lazar N.A. (2016). The ASA’s statement on p-values: Context, process, and purpose. Am. Stat..

[72] Sullivan G.M., Feinn R. (2012). Using effect size—Or why the P value is not enough. J. Grad. Med. Educ..

[73] Yates F. (1934). Contingency tables involving small numbers and the χ 2 test. Suppl. J. R. Stat. Soc..

[74] Coe R., Merino C. (2003). Magnitud del efecto: Una guía para investigadores y usuarios. Rev. Psicol..

[75] Chinn S. (2000). A simple method for converting an odds ratio to effect size for use in meta-analysis. Stat. Med..

[76] Dryglas D., Różycki P. (2016). European spa resorts in the perception of non-commercial and commercial patients and tourists: The case study of Poland. E-Rev. Tour. Res..

[77] Hatzichristos T. (2004). Delineation of demographic regions with GIS and computational intelligence. Environ. Plan. B Plan. Des..

[78] Kaski S., Kohonen T. (1996). Exploratory data analysis by the Self-Organizing Map: Structures of welfare and poverty in the world. Neural Networks in Financial Engineering-Proceedings of the Third International Conference on Neural Networks in the Capital Markets.

[79] Cottrell M., Letrémy P. (2005). How to use the Kohonen algorithm to simultaneously analyze individuals and modalities in a survey. Neurocomputing.

[80] Hamaina R., Leduc T., Moreau G., Gensel J. (2012). Towards urban fabrics characterization based on buildings footprints. Bridging the Geographic Information Sciences.

[81] Bação F., Lobo V., Painho M. (2005). Self-organizing maps as substitutes for k-means clustering. Comput. Sci.–ICCS.

[82] Yan J., Thill J.C. (2009). Visual data mining in spatial interaction analysis with self-organizing maps. Environ. Plan. B Plan. Des..

[83] Lagus K., Vatanen T., Kettunen O., Heikkil A., Heikkil M., Pantzar M., Honkela T. (2013). Paths of wellbeing on self-organizing maps. Adv. Intell. Syst. Comput..

[84] Trafialek J., Laskowski W., Kolanowski W. (2015). The use of Kohonen’s artificial neural networks for analyzing the results of HACCP system declarative survey. Food Control.

[85] Voutilainen A., Kvist T., Sherwood P.R., Vehviläinen-Julkunen K. (2014). A new look at patient satisfaction: Learning from self-organizing maps. Nurs. Res..

[86] Behnisch M., Ultsch A. (2009). Urban data-mining: Spatiotemporal exploration of multidimensional data. Build. Res. Inf..

[87] Kauko T. (2005). Using the self-organising map to identify regularities across country-specific housing-market contexts. Environ. Plan. B Plan. Des..

